# Effect of Selected Environmental Factors on the Microbicidal Effectiveness of Radiant Catalytic Ionization

**DOI:** 10.3389/fmicb.2019.03057

**Published:** 2020-01-23

**Authors:** Krzysztof Skowron, Ewa Wałecka-Zacharska, Katarzyna Grudlewska, Joanna Kwiecińska-Piróg, Natalia Wiktorczyk, Maria Kowalska, Zbigniew Paluszak, Katarzyna Kosek-Paszkowska, Klaudia Brożek, Jakub Korkus, Eugenia Gospodarek-Komkowska

**Affiliations:** ^1^Department of Microbiology, Collegium Medicum of L. Rydygier in Bydgoszcz, Nicolaus Copernicus University in Toruñ, Bydgoszcz, Poland; ^2^Department of Food Hygiene and Consumer Health, Wrocław University of Environmental and Life Sciences, Wrocław, Poland; ^3^Department of Food Analytics and Environmental Protection, Faculty of Chemical Technology and Engineering, UTP University of Sciences and Technology in Bydgoszcz, Bydgoszcz, Poland; ^4^Department of Microbiology and Food Technology, UTP University of Sciences and Technology in Bydgoszcz, Bydgoszcz, Poland

**Keywords:** radiant catalytic ionization, stainless steel, rubber, foodborne pathogens, microbicidal effectiveness

## Abstract

The aim of this study was the assessment of the effect of time exposure, temperature, distance, and organic contaminants on radiant catalytic ionization (RCI) microbicidal effectiveness. The number of all examined bacteria decreased together with time exposure of RCI. The lowest recovery was obtained, both from the rubber surface (6.36 log CFU × cm^–2^) and steel (6.04 log CFU × cm^–2^) in the case of *Escherichia coli* O157:H7. On the other hand, *Staphylococcus aureus* was isolated in the largest number (rubber: 7.88 log CFU × cm^–2^, steel: 7.79 log CFU × cm^–2^). Among the tested environmental conditions, the greatest bacterial population was re-isolated at 4°C (distance: 0.5 m, time: 24 h), whereas the lowest population was found at a distance of 0.5 m (temperature: 20°C, time: 24 h) and on surfaces without contamination. In the samples treated with RCI, the bacterial population was the lowest on non-contaminated surfaces, ranging from 3.76 log CFU × cm^–2^ (*E. coli* O157:H7) to 5.58 log CFU × cm^–2^ (*S. aureus*) for the rubber, and from 3.26 log CFU × cm^–2^ (*E. coli* O157:H7) to 5.20 log CFU × cm^–2^ (*S. aureus*) for the stainless steel. The highest bacteria number was isolated from surfaces contaminated with meat and fish pulp. The lowest bacterial reduction caused by RCI was found in the case of rubber contaminated with meat-fish pulp (24 h, 0.5 m, 20°C). The reduction rate was equal to 0.89 log CFU × cm^–2^ for *S. aureus*, 1.17 log CFU × cm^–2^ for *Listeria monocytogenes*, 1.43 log CFU × cm^–2^ for *Salmonella* Enteritidis and 1.61 log CFU × cm^–2^ for *E. coli* O157:H7. In turn, the greatest bacterial reduction was found in the case of non-contaminated steel (24 h, 0.5 m, 37°C). The reduction rate was equal to 4.52 log CFU × cm^–2^ for *L. monocytogenes*, 3.61 log CFU × cm^–2^ for *S.* Enteritidis, 2.98 log CFU × cm^–2^ for *E. coli* O157:H7 and 2.77 log CFU × cm^–2^ for *S. aureus*. RCI allows the inactivation of pathogens from stainless steel and rubber surfaces. Its efficacy is species-dependent and affected by environmental factors.

## Introduction

Over recent years, a high degree of final product contamination with pathogenic bacteria e.g., *Listeria* spp., *Salmonella* spp., *Campylobacter* spp., *Escherichia* spp. has been reported in the food industry (Kathariou, 2002; European Food Safety Authority [EFSA], 2017). One of the main reasons behind food products contamination in food plants is bacterial biofilm on the food-processing surfaces (tanks, tapes, cutting elements), ceilings, and walls of production halls (Pan et al., 2006; Swaminathan and Gerner-Smidt, 2007). Such biofilms are difficult to eliminate and pose a risk to consumer health. Nowadays, there are many methods used for the eradication of bacteria i.e., chemical disinfection, pasteurization, sonication, radioactive radiation, and high-pressure treatment. Nonetheless, there is still a need for new disinfection methods that are safe for the consumer and allow the elimination of both the planktonic form of bacteria and biofilms (Walczycka, 2005; Nowicka et al., 2014). One alternative method is the use of heterogenous photocatalytic oxidation. The first work on this subject described its use mainly for wastewater treatment (Zhao and Yang, 2003). In the 1990s this technology was developed by the National Aeronautics and Space Administration (NASA) for air purification. After technology optimization, it was defined as Radiant Catalytic Ionization (RCI) (Space Fundation, Radiant Catalytic Ionization Air & Water Purification, 2014). The RCI cell consists of matrices of elongated polycarbonate components, arranged in a parallel orientation resembling a honeycomb. A coating of matrices comprises a grouping of the materials: titanium dioxide, rhodium, silver, and copper. On the opposite site a broad-spectrum UV light source is located. The UV lamp utilizes argon gas with mercury and carbide filaments with a spectrum of 100 and 367 nm (Grinshpun et al., 2007; Małecka and Borowski, 2011). Titanium dioxide, emitted by the apparatus, acts as a photocatalyst and was approved by the FDA (Food and Drug Administration) as non-toxic for use in the food, pharmacy, and cosmetics industries (Yemmireddy and Hung, 2015). The photocatalytic activity of TiO_2_ is associated with its crystal structure, which is associated with a characteristic band gap (Foster et al., 2011; Llorensa et al., 2012). There are three main polyforms of titanium dioxide: anatase, rutile, and brookite, of which anatase is the most effective photocatalyst (Miyagi et al., 2004; Foster et al., 2011). TiO_2_ nanoparticles are most often produced using the sol-gel method (Foster et al., 2011; Llorensa et al., 2012). TiO_2_ nanoparticles (anatase or rutile) were also attached to cellulose (Daoud et al., 2005). In the case of RCI cells, we deal with TiO_2_ nanotubes generated in the hydrothermal reaction of titanium oxide with NaOH at 180°C (Llorensa et al., 2012). Titanium dioxide is semi-conductive and a photon adsorption promotes valence electrons (e_vb_-) to the conduction band (e_cb_-), leaving positively charged space in the valence band (h_vb_+). The energy required for electron promotion is about 3.2 eV, indicating that photolysis might be activated by photons with a 385 nm wavelength e.g., UV-A UV radiation with energy higher than the energy of the TiO_2_ crystal band (3.2 eV for anatase, 3.03 eV for rutile) is absorbed by the catalyst, then the electron is promoted to the conduction band, creating a pair of negatively charged free electrons and a positive charge of the electron hole (Miyagi et al., 2004). Free electrons might migrate within the hole of the conduction band, which can be filled with electrons of adjacent molecules as well. The hole of the valence band and electrons may recombine (mass recombination) as an unproductive reaction, or react with each other after reaching the surface, releasing reactive oxygen species (ROS) i.e., O_2_^–^ and OH. In turn, ROS react in the solution, producing hydroxyl and hydroperoxyl radicals (Foster et al., 2011; Llorensa et al., 2012). It has been found that RCI also generates a small amount of ozone (Grinshpun et al., 2007; Małecka and Borowski, 2011). A schematic diagram of the chain oxidation reaction is shown below (Chong et al., 2010):

(1) TiO_2_ + hυ→ e^–^ + h^+^
(2) e^–^_CB_ → e^–^_TR_
(3) h^+^ υ_B_ → h^+^_TR_
(4) e^–^_TR_ + h^+^_υB_ (h^+^_TR_) → e^–^_CB_ + heat
(5) (O_2_)_ads_ + e^–^ → O_2_^–^ ⋅
(6) OH^–^ + h^+^ →⋅ OH
(7) ⋅ OH: R – H + ⋅ OH → R′⋅ H_2_O

Photocatalysis has been found to not only effectively eliminate Gram-positive and Gram-negative bacteria, spores, viruses, fungi, and protozoa, but also inactivate prions and bacterial toxins (Paspaltsis et al., 2006; Foster et al., 2011). Gram-positive bacteria have been shown to be more resistant to photocatalytic disinfection than Gram-negative (Pal et al., 2005, 2007). Killing of the bacterial cell is associated with the oxidation of coenzyme A molecules, the oxidation of unsaturated phospholipids and outer cell membrane damage (Grinshpun et al., 2007). Viruses are destroyed by genetic material damage and the impairment of capsid protein functionality (Grinshpun et al., 2007). RCI is applied mainly in the air purification industry (Winczewski and Malicki, 2011). Lately, this method was used for the elimination of pathogens from stainless steel, indicating the possibility of its application for the disinfection of food-processing surfaces and health care facilities (Ortega et al., 2007; Chorianopoulos et al., 2011).

The aim of this study was the assessment of RCI efficacy as a disinfection method against selected pathogens (*Listeria monocytogenes* ATCC^®^ 19111^TM^, *Escherichia coli* O157:H7 ATCC^®^ 43895^TM^, *Staphylococcus aureus* ATCC^®^ 29213^TM^, *Salmonella* Enteritidis ATCC^®^ 13076^TM^) on stainless steel and rubber. The effect of time exposure, temperature, distance from the apparatus, and the presence of organic contaminants was evaluated.

## Materials and Methods

### Materials

The study was conducted on four strains purchased from the American Tissue Culture Collection (ATCC^®^): *E. coli* O157:H7 (ATCC^®^ 43895^TM^), *L. monocytogenes* (ATCC^®^ 19111^TM^), *S.* Enteritidis ATCC^®^ 13076^TM^, and *S. aureus* ATCC^®^ 29213^TM^.

### Assessment of Ozone Concentration in the Air Generated by ActivTek

Samples were collected using the aspiration method that absorbs ozone and formaldehyde in distilled water. Since formaldehyde and nitrogen oxides are present in the atmospheric air and interfere with ozone determination, their content has been taken into account. The test was carried out in a hermetic, sterile steel chamber with a volume of 1.4 m^3^. Quantitative analysis was made with the colorimetric method using rosaniline hydrochloride (Polska Norma Pn-Z-04007-05, 1986). Ozone detection was based on the reaction with eugenol (4-allilo-2-methoxyphenol). Ozone cleaves the double bond of eugenol, producing formaldehyde in the amount equivalent to the ozone concentration. In turn, the aqueous solution of formaldehyde reacts with the sodium dichloro-sulfito-mercurate II solution and rosaniline hydrochloride, forming a color complex, the intensity of which is proportional to the formaldehyde concentration. Samples were collected according to the Polish Norm PN-Z-04008-7, 2002 (Chapter 5) (Polska Norma Pn-Z-04008-7, 2002). Sixty liters of the air were taken for analysis, which was done immediately after sampling. The ozone concentration was determined at different temperatures (4°C, 20°C, 37°C), distances from the apparatus (0, 0.5, 1, 2 m) and RCI exposure time (1, 6, 12 h). The validation parameters were: variation coefficient 5.1%, limit of quantification 0.003 mg, linear correlation coefficient 0.9963.

### Preparation of Surfaces

Two types of surfaces, i.e., stainless steel AISI 304 and natural rubber, most commonly applied in the food industry, were used in the study. Fragments of rubber and steel (1 cm × 2 cm) were cut using hydroabrasive technology. The fragments were washed with 70% ethanol, rinsed with deionized water, dried, and sterilized using high energy electron beam irradiation (25 kGy).

### Preparation of Bacterial Suspension and Surface Contamination

For each strain, a suspension of 0.5 McFarland’s scale [*S.* Enteritidis 1.12 × 10^8^ (±3.51 × 10^6^) CFU × cm^–3^; *S. aureus* 1.72 × 10^8^ (±4.17 × 10^7^) CFU × cm^–3^; *E. coli* 5.20 × 10^8^ (±2.86 × 10^7^) CFU × cm^–3^; *L. monocytogenes* 7.80 × 10^7^ (±1.66 × 10^7^) CFU × cm^–3^] in sterile saline was prepared. Suspension of 50 μl was applied on sterile fragments of the examined surfaces to allow the adhesion of planktonic forms of bacteria. The fragments were dried in the laminar air flow chamber for 2 h. After this time, the prepared surfaces were immediately used for testing. The number of bacteria re-isolated from the surface at 0 h of RCI activity (initial number) was determined as described in the section “Determination of Bacteria Number Recovered From the Treated Surface.”

Based on the results obtained for all tested bacteria used in the study, the initial adhesion coefficient was calculated according to the following formula:

$$\mathrm{IAC}\left[\%\right]=\frac{a}{b}\times 100$$

where:

IAC – initial adhesion coefficient [%],
*a* – bacterial count re-isolated from the surface of 1 cm^2^ after adhesion and drying [CFU],
*b* – bacterial count re-isolated from the suspension of 1 cm^3^ [CFU].

### Preparation of Bacterial Suspension and Surface Contamination With Pulp

For each strain, a suspension of 1.0 McFarland’s scale [*S.* Enteritidis 7.50 × 10^13^ (± 2.39 × 10^12^) CFU × cm^–3^; *S. aureus* 3.80 × 10^14^ (± 1.07 × 10^14^) CFU × cm^–3^; *E. coli* 6.50 × 10^14^ (± 9.70 × 10^13^) CFU × cm^–3^; *L. monocytogenes* 1.30 × 10^13^ (± 8.45 × 10^12^) CFU × cm^–3^] in sterile saline was prepared. The suspension was used to assess the effect of contamination with organic pulp on RCI efficacy. The microbial suspensions were mixed in a volumetric ratio (v/v) 1: 1 (1 cm^3^ *E. coli*, 1 cm^3^ *L. monocytogenes*, 1 cm^3^ *S. aureus*, 1 cm^3^ *S.* Enteritidis). The aim of making a mixture of bacterial suspensions was to reflect, as accurately as possible, the real conditions prevailing in the natural environment where contamination with different species of micro-organisms simultaneously occurs.

### Assessment of Factors Affecting Bactericidal Effectiveness of RCI Technology

The effect of time exposure, temperature of the environment, and distance from the instrument on bactericidal efficacy of RCI was investigated. The fragments contaminated with bacteria were placed in an open Petri dish and exposed to RCI. RCI was generated using Induct 750 apparatus (ActivTek). Each fragment was contaminated with only one bacterial species. Each experiment was conducted in triplicate.

The RCI cell has a structure optimized for purified air flow. A detailed description of the construction and operation of the Induct 750 device was presented by Skowron et al. (2018a).

#### Effect of Time Exposure on Bactericidal Efficacy of RCI

The fragments contaminated with bacteria were placed at 20°C (Faster BH-EN 2004), at a distance of 0.5 m from the Induct 750 (ActivTek) apparatus and RCI-treated for 1, 6, 12, and 24 h. The number of bacteria re-isolated after 24 h from the surfaces not exposed to RCI was also evaluated (positive control).

Moreover, a bactericidal additional effect of the RCI technology was established in relation to the drying effect of bacteria attached on the tested surfaces after 24 h. The above absolute coefficient of RCI effectiveness was calculated as:

$$A\text{EC}\left[\%\right]=\frac{{\text{RCI}}_{24\,\text{h}}}{\text{K}\,{\left(+\right)}_{24\,\text{h}}}\times 100$$

where:

AEC – absolute coefficient of RCI effectiveness [%],
K(+)_24 *h*_ – number of bacteria isolated from the positive control not exposed to RCI after 24 h [CFU × cm^–2^],
RCI_24__*h*_ – number of bacteria isolated from the samples treated with RCI for 24 h [CFU × cm^–2^].

#### Effect of Temperature of the Environment on Bactericidal Efficacy of RCI

The contaminated fragments were placed at 4°C (refrigerator Aged LKPv1420), 20°C (Faster BH-EN 2004), and 37°C (incubator CLW1000 TOP + INOX/G), at a distance of 0.5 m from the apparatus, and RCI was applied for 24 h. The temperature was monitored every 1 min during the experiment using a radio temperature monitoring system Boomerang (ICU Scandinavian). The positive controls were contaminated fragments incubated at the appropriate temperature for 24 h but not RCI-treated.

#### Effect of the Distance From Induct 750 Apparatus on Bactericidal Efficacy of RCI

The contaminated fragments were placed at 20°C (Faster BH-EN 2004), at a distance of 0, 0.5, 1, and 2 m from the apparatus. The RCI was applied for 24 h.

#### Effect of the Presence of Organic Contaminants on Bactericidal Efficacy of RCI

Three types of pulp were used: meat-fish, milk-cheese, and vegetable. The pulp used in the study was prepared as a mixture of appropriate food products with sterile physiological saline. Prepared masses of minced poultry meat, salmon filet, soft cheese, milk, and carrots were sterilized by radiation at the Institute of Nuclear Chemistry and Technology in Warsaw (Poland) using the ELEKTRONIKA 10/10 microwave accelerator. Carrots were ground into small pieces before sterilization and homogenization. To prepare test pulps, appropriate food products were placed in a bag and sterile saline was added, followed by placing the bag in a BagMixer^®^ 400 VW homogenizer (Interscience, France) and homogenizing for 10 min at room temperature. To prepare the meat-fish pulp, 50 g of minced poultry meat was mixed with 50 g of salmon filet and 100 ml of sterile saline was added. The cheese-milk pulp was prepared by mixing 100 g of soft cheese, 50 ml of milk, and 50 ml of sterile saline. To prepare the vegetable pulp, 100 g of grounded carrot was mixed with 60 ml of sterile saline. The weights and volumes of ingredients were selected in such a way as to maximally ensure the similar consistency of the obtained pulp. The finished pulp was stored in bags for a maximum of 24 h at 4°C.

Bacterial suspension of 1.0 McF was mixed with an equal volume of selected pulp (4 cm^3:^ 4 cm^3^). Suspension of 50 μl was applied on sterile fragments of the examined surfaces. The fragments contaminated with such a mixture were dried and placed at 20°C (Faster BH-EN 2004) at a distance of 0.5 m from the instrument, and exposed to RCI for 24 h. The positive controls were the fragments contaminated with bacteria and pulp not treated with RCI.

### Determination of Bacteria Number Recovered From the Treated Surface

The fragments of rubber or steel from different environmental conditions were placed in sterile PBS (20 ml), sonicated (Ultrasonic DU-4, Nickel-Electro) for 10 min (30 kHz, 150 W), and shaken for 5 min at 400 rpm. The obtained suspensions were left to rest for about 30 min at room temperature before the preparation of further dilutions in order to allow the resuscitation of microorganisms (PHE Microbiology Services, 2014). Then, serial 10-fold dilutions were done and plated onto MacConkey agar (Becton-Dickinson) (*E. coli*), Oxford agar (Oxoid) (*L. monocytogenes*), XLD agar (Merck) (*S.* Enteritidis), or Chapman agar (Becton-Dickinson) (*S. aureus*) and incubated at 37°C for 24 h. The number of bacteria re-isolated from the contaminated surface was expressed as log CFU × cm^–2^.

As a mixture of suspensions of the tested bacterial species was used during the assessment of the impact of organic contamination on the antimicrobial effectiveness of RCI, selective media were applied to distinguish the recovered bacteria. In order to ensure comparable results between different tested conditions, selective media were used in all experiments including these assessing the effectiveness of RCI against individual bacterial species.

Due to the use of selective media, it was decided to evaluate and compare the recovery of the tested bacteria for a non-selective medium (Trypticase soy agar, TSA) and a selective medium suitable for the species. For this purpose, steel fragments with attached bacteria were prepared. One set was stored for 24 h at 20°C without RCI (K+) and the other one was for 24 h exposed to RCI at a distance of 0.5 m at 20°C. Then, the bacteria were re-isolated from the tested surfaces and counted according to the procedure described above.

### Statistical Analysis

Each experiment was repeated three times. The Tukey *post hoc* test was performed with Statistica 12.0 PL software (StatSoft) to determine whether statistical differences existed between different experimental groups. Significance was set at *p* level of < 0.05.

## Results

### Assessment of Ozone Concentration in the Air Generated During RCI

**FIGURE 1:**
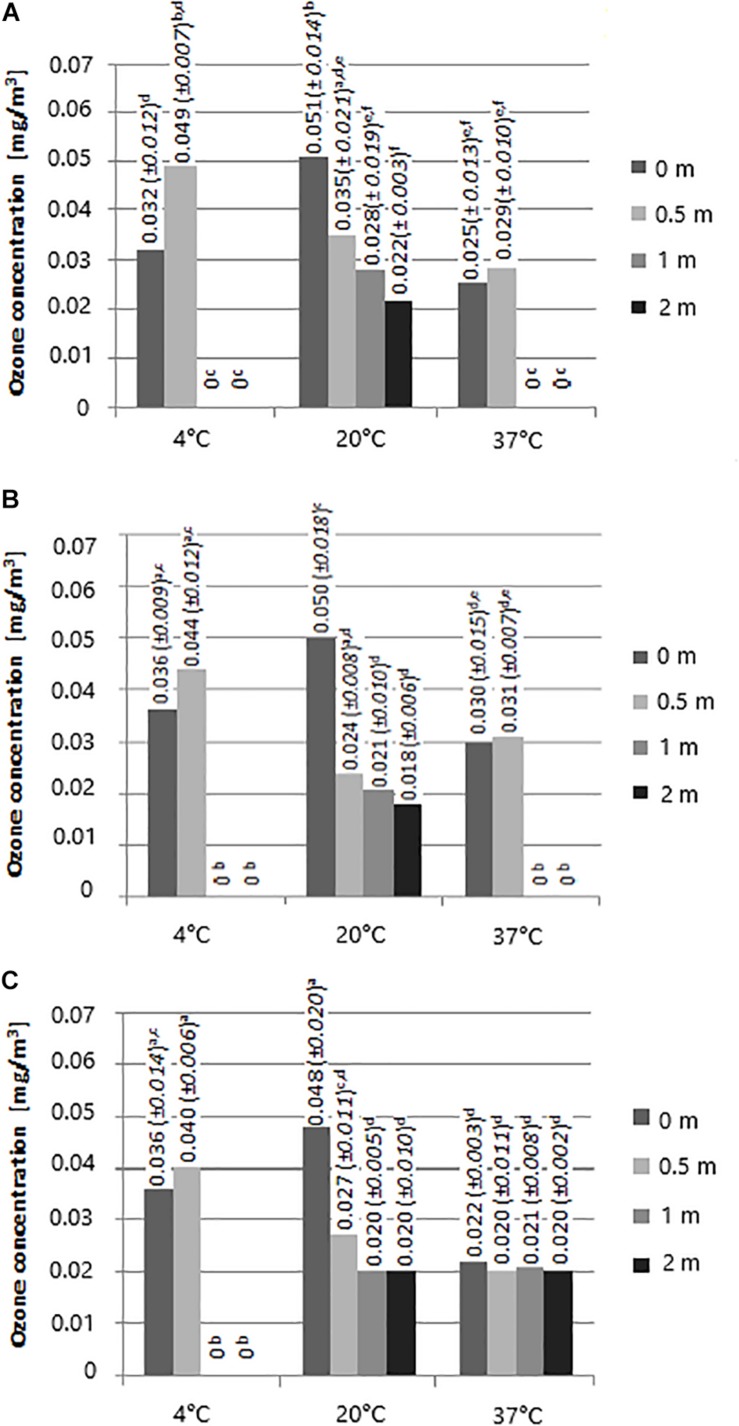
The concentration of ozone in the air during operation of the RCI device for **(A)** 1 h, **(B)** 6 h, **(C)** 12 h depending on the temperature and distance from the source [a, b, c – values marked with different letters are significantly different (*P* < 0.05)].

The highest ozone concentration was produced at 20°C at 0 m distance from the ActivTek instrument, regardless of time exposure (Figure 1). High ozone content was also detected at 4°C at a 0.5 m distance from the instrument (1 h – 0.049 mg/m^3^, 6 h – 0.44 mg/m^3^, 12 h – 0.04 mg/m^3^). Ozone was detected in each variant at 20°C, whereas this was not observed at a distance of 1 and 2 m at 4°C, irrespective of time exposure, and at 37°C after 1 and 6 h.

### Evaluation of Bacterial Recovery After Using Selective and Non-selective Media

The results of this analysis are given in Table 1. Bacterial recovery was slightly higher when using non-selective media, however, there were no statistically significant differences in growth on TSA and selective media. In addition, statistically significant differences between strains exposed to RCI and control variants were found for both types of media for all the tested species.

**TABLE 1:** The number of bacteria re-isolated from a steel surface and cultivated on various media.

**Experiment variant**	**Number of bacteria [log CFU × cm^–2^]**	
	***Escherichia coli* O157:H7**	
	**TSA**	**MacConkey agar**

K(+)	5.70 (±0.94)^a^	4.80 (±1.03)^a^
RCI	4.13 (±2.80)^b^	3.08 (±0.16)^b^

	***Listeria monocytogenes***	

	**TSA**	**OXFORD**

K(+)	6.90 (±0.82)^a^	6.50 (±0.20)^a^
RCI	3.70 (±1.03)^b^	3.00 (±0.65)^b^

	***Staphylococcus aureus***	

	**TSA**	**Chapman agar**

K(+)	7.60 (±3.94)^a^	6.94 (±4.27)^a^
RCI	6.19 (±2.80)^b^	5.40 (±4.16)^b^

	***Salmonella* Enteritidis**	

	**TSA**	**XLD**

K(+)	6.50 (±2.16)^a^	5.40 (±1.54)^a^
RCI	4.41 (±1.32)^b^	3.37 (±0.52)^b^

*a,b,c,… – values marked with different letters in the same row and column differ statistically significantly for a particular bacteria.*

### Adhesion of Bacteria to Stainless Steel and Rubber Surfaces

**FIGURE 2:**
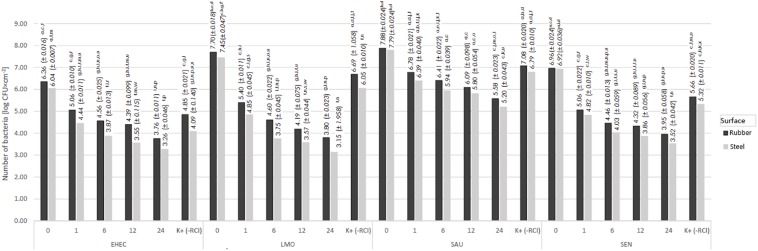
Changes in the number of bacteria under the influence of RCI depending on the exposure time [a, b, c – values marked with different letters are significantly different (*P* < 0.05); EHEC – *E. coli* O157:H7; LMO – *L. monocytogenes*; SAU – *S. aureus*; SEN – *S*. Enteritidis].

**FIGURE 3:**
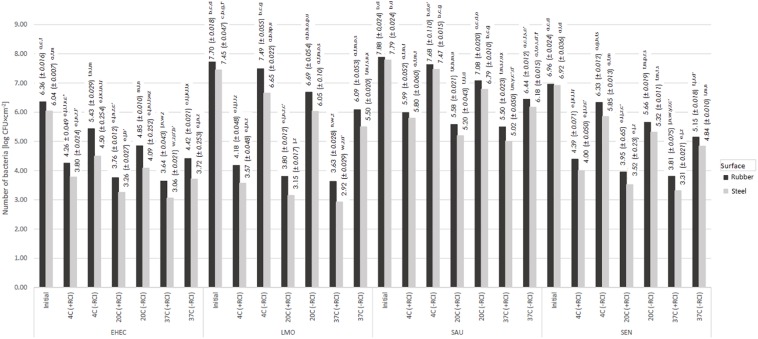
Changes in the number of bacteria under the influence of RCI depending on the temperature [a, b, c – values marked with different letters are significantly different (*P* < 0.05); EHEC – *E. coli* O157:H7; LMO – *L. monocytogenes*; SAU – *S. aureus*; SEN – *S*. Enteritidis].

**FIGURE 4:**
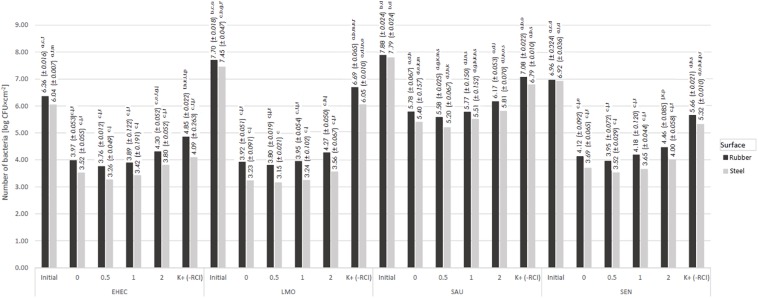
Changes in the number of bacteria under the influence of RCI depending on the distance from device [a, b, c – values marked with different letters are significantly different (*P* < 0.05); EHEC – *E. coli* O157:H7; LMO – *L. monocytogenes*; SAU – *S. aureus*; SEN – *S*. Enteritidis].

**FIGURE 5:**
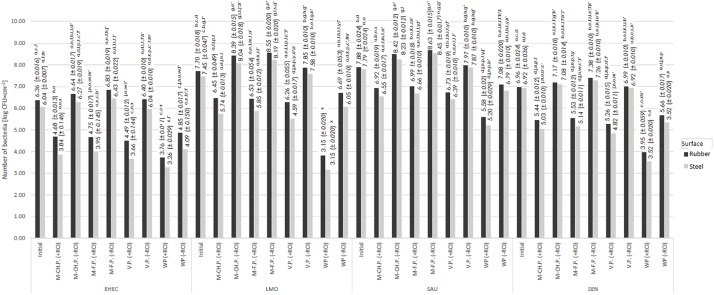
Changes in the number of bacteria under the influence of RCI depending on the organic pollution [M-CH.P. – milk-cheese pulp, M-F.P. – meat-fish pulp, V.P. – vegetable pulp, WP – without pulp, +RCI – with RCI, −RCI – without RCI; a, b, c – values marked with different letters are significantly different (*P* < 0.05); EHEC – *E. coli* O157:H7; LMO – *L. monocytogenes*; SAU – *S. aureus*; SEN – *S*. Enteritidis].

The number of recovered bacteria was species and surface-dependent. Greater bacteria recovery was found for the rubber than the steel surface. The observed differences were in most cases not statistically significant (Figures 2–5). The lowest number was re-isolated for *E. coli* on the rubber surface (6.36 log CFU × cm^–2^) and stainless steel (6.04 log CFU × cm^–2^) (Figures 2–5). The greatest bacteria number was noted for *S. aureus*, and was 7.88 log CFU × cm^–2^ and 7.79 log CFU × cm^–2^ for rubber and steel, respectively.

The initial adhesion coefficient calculated for all tested bacterial species and both tested surfaces are presented in Table 2.

**TABLE 2:** Initial adhesion coefficient (IAC) of tested bacterial species.

**Species**	**IAC rubber (%)**	**IAC steel (%)**
*E. coli*	73.0	69.3
*L. monocytogenes*	97.6	94.4
*S. aureus*	95.7	94.6
*S.* Enteritidis	86.5	86.0

### Effect of Time Exposure on Bactericidal Efficacy of RCI

**FIGURE 6:**
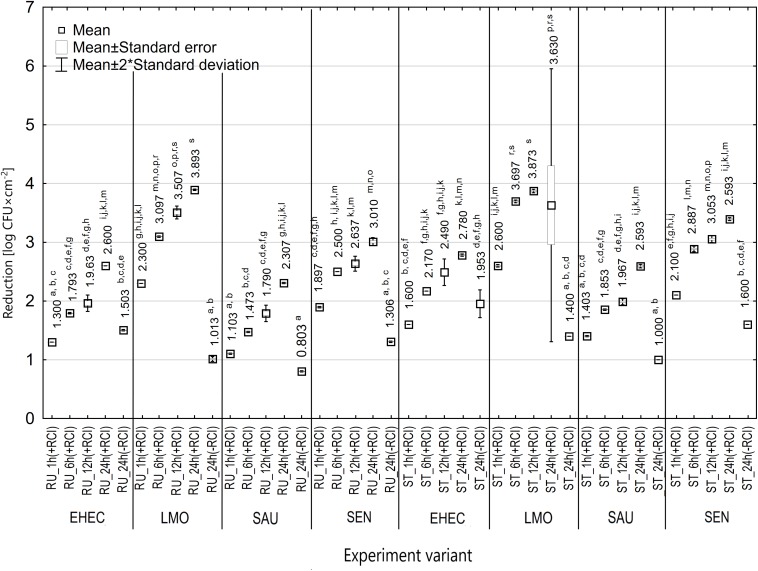
Reduction in the number of bacteria under the influence of RCI depending on the exposure time [RU – rubber, ST – stainless steel; a, b, c – values marked with different letters are significantly different (*P* < 0.05); EHEC – *E. coli* O157:H7; LMO – *L. monocytogenes*; SAU – *S. aureus*; SEN – *S*. Enteritidis].

It was shown that time exposure influenced the bactericidal activity of RCI. The number of bacteria decreased along with time exposure, ranging from 3.76 log CFU × cm^–2^ (*E. coli*) to 5.58 log CFU × cm^–2^ (*S. aureus*) for the rubber surface, and from 3.26 log CFU × cm^–2^ (*E. coli*) to 5.20 log CFU × cm^–2^ (*S. aureus*) for the steel surface (Figure 2). The number of recovered bacteria after 24 h of the experiment was significantly lower compared to the control variant (Figure 2). The calculated bactericidal effect of the RCI technology after 24 h exposure is shown in Table 3. Significant differences were found also after 1 h treatment for *L. monocytogenes* and after 6 h for *S. aureus* and *S*. Enteritidis. The most resistant to RCI was *S. aureus* with the reduction number of 2.31 log CFU × cm^–2^ for the rubber and 2.59 log CFU × cm^–2^ for the steel (Figure 6).

**TABLE 3:** Absolute coefficient of RCI effectiveness (AEC) (%)

**Species**	**AEC rubber**	**AEC steel**
*E. coli* O157:H7	78.0 (±1.12)^∗^	80.0 (±2.73)
*L. monocytogenes*	57.0 (±13.60)	52.0 (±2.16)
*S. aureus*	79.0 (±2.37)	77.0 (±3.62)
*S.* Enteritidis	70.0 (±2.91)	66.0 (±2.18)

*^∗^Standard deviation.*

### Effect of Temperature of the Environment on Bactericidal Efficacy of RCI

**FIGURE 7:**
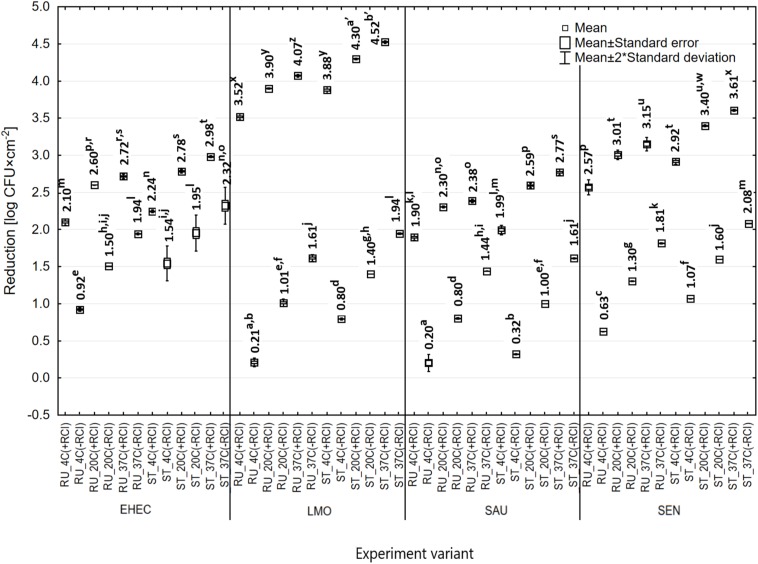
Reduction in the number of bacteria under the influence of RCI depending on the temperature [RU – rubber, ST – stainless steel; a, b, c – values marked with different letters are significantly different (*P* < 0.05); EHEC – *E. coli* O157:H7; LMO – *L. monocytogenes*; SAU – *S. aureus*; SEN – *S*. Enteritidis].

The number of bacteria subjected to RCI was significantly lower compared to the control, irrespective of temperature (Figure 7). The bactericidal efficacy of RCI increased together with temperature. The reduction of bacteria numbers after 24 h of RCI exposure at 4°C ranged from 1.90 log CFU × cm^–2^ (*S. aureus*) to 3.52 log CFU × cm^–2^ (*L. monocytogenes*) for the rubber, and from 1.99 log CFU × cm^–2^ (*S. aureus*) to 3.88 log CFU × cm^–2^ (*L. monocytogenes*) for the steel. In turn, the reduction of bacteria numbers at 37°C was statistically significantly higher and ranged from 2.38 log CFU × cm^–2^ (*S. aureus*) to 4.07 log CFU × cm^–2^ (*L. monocytogenes*) for the rubber, and from 2.77 log CFU × cm^–2^ (*S. aureus*) to 4.52 log CFU × cm^–2^ (*L. monocytogenes*) for the steel. The RCI more effectively eliminated bacteria from the stainless steel surface than from the rubber. The use of RCI technology resulted in statistically significantly higher decreases in the number of bacteria compared to the control variant in the case of the same strain applied to the same surface under given temperature conditions (Figure 7). The most susceptible to RCI treatment was *L. monocytogenes*, while *S. aureus* was the most resistant.

### Effect of Distance From the Induct 750 Apparatus on Bactericidal Efficacy of RCI

**FIGURE 8:**
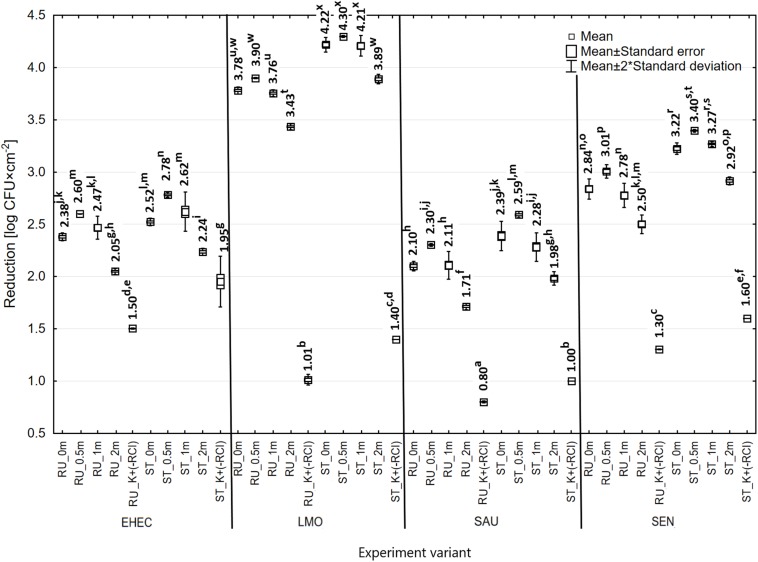
Reduction in the number of bacteria under the influence of RCI depending on the distance from device [RU – rubber, ST – stainless steel; a, b, c – values marked with different letters are significantly different (*P* < 0.05); EHEC – *E. coli* O157:H7; LMO – *L. monocytogenes*; SAU – *S. aureus*; SEN – *S*. Enteritidis].

The reduction of bacteria number after 24 h of RCI treatment was significantly higher compared to the control bacteria, regardless of the distance from the Induct 750 instrument (Figures 4, 8). The lowest number of bacteria was re-isolated from surfaces placed at a distance of 0.5 m from the instrument and ranged from 3.76 log CFU × cm^–2^ (*E. coli*) to 5.58 log CFU × cm^–2^ (*S. aureus*) for the rubber, and from 3.26 log CFU × cm^–2^ (*E. coli*) to 5.20 log CFU × cm^–2^ (*S. aureus*) for the steel. On the other hand, the lowest bactericidal effect of RCI was observed at a distance of 2 m from the instrument. Irrespective of the distance, RCI was more efficacious against the steel surfaces.

### Effect of the Presence of Organic Contaminants on Bactericidal Efficacy of RCI

**FIGURE 9:**
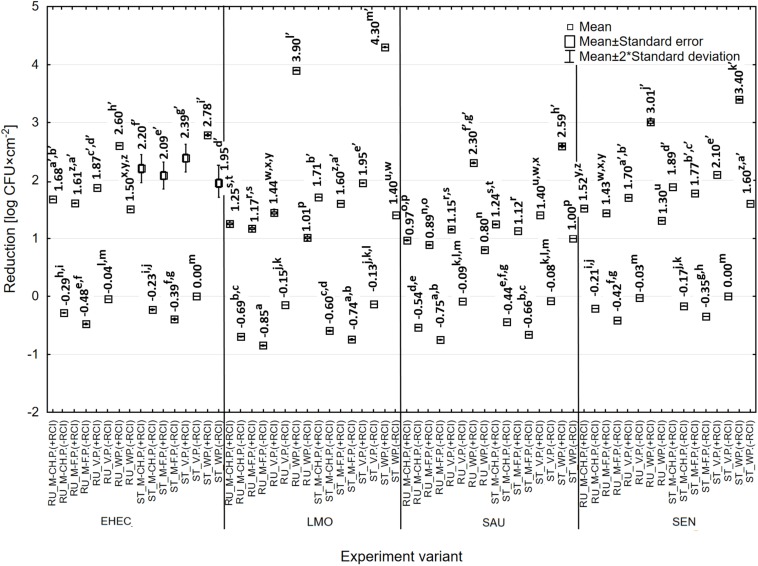
Reduction in the number of bacteria under the influence of RCI depending on the organic pollution [RU – rubber, ST – stainless steel, M-CH.P. – milk-cheese pulp, M-F.P. – meat-fish pulp, V.P. – vegetable pulp, WP – without pulp, +RCI – with RCI, -RCI – without RCI; a, b, c – values marked with different letters are significantly different (*P* < 0.05); EHEC – *E. coli* O157:H7; LMO – *L. monocytogenes*; SAU – *S. aureus*; SEN – *S*. Enteritidis].

The number of bacteria treated and non-treated with RCI, recovered from the surfaces, was affected by the presence and type of organic contaminants. The lowest bacterial recovery was noted for contaminants-free surfaces and ranged from 3.76 log CFU × cm^–2^ (*E. coli*) to 5.58 log CFU × cm^–2^ (*S. aureus*) for the rubber and from 3.26 log CFU × cm^–2^ (*E. coli*) to 5.20 log CFU × cm^–2^ (*S. aureus*) for the steel (Figure 5). In turn, the greatest number of bacteria was isolated from the surfaces contaminated with meat-fish pulp. The reduction of bacteria number ranged from 0.97 log CFU × cm^–2^ (*S. aureus*) to 1.61 log CFU × cm^–2^ (*E. coli*) and from 1.12 log CFU × cm^–2^ (*S. aureus*) to 2.09 log CFU × cm^–2^ (*E. coli*) for the rubber and steel, respectively (Figure 9). Vegetable pulp had the lowest impact on bactericidal efficacy of RCI.

## Discussion

The increasing number of food-borne diseases has determined the necessity of searching for new, safe, and effective disinfection methods, allowing the eradication of both planktonic cells and biofilm (Walczycka, 2005; Nowicka et al., 2014; European Food Safety Authority [EFSA], 2017). RCI was reported to be an effective method of elimination of microbiological contaminants, including viruses, vegetative forms, and spores of bacteria, from the surfaces (Matsunaga et al., 1985; Gogniat et al., 2006; Paspaltsis et al., 2006; Skowron et al., 2018a, b) and air (Grinshpun et al., 2007). It has been found that this technology can be successfully applied in many industries. However, there is no data on the impact of various environmental factors on the antimicrobial activity of RCI technology.

In this study we applied RCI to eradicate pathogens from the surfaces used in food plants (stainless steel and rubber). The effect of temperature of the environment, distance from the apparatus, time exposure, and presence of organic contaminants on RCI efficacy was evaluated. RCI effectiveness was associated with species and surface type. This supports our previous studies (Skowron et al., 2018a, b, 2019). We showed recently that RCI inactivated bacteria most efficiently on a varnished veneer surface and steel ASI 304, while the lowest reduction was observed for a glazed earthenware and polyamide surface. The least sensitive was *S. aureus*, whereas *Acinetobacter baumannii* was the most susceptible (Skowron et al., 2018b). In the latest study assessing the RCI efficacy against *Klebsiella pneumoniae* NDM from surfaces used for the production of bedding and mattresses in hospitals, we found the highest efficiency of the method against PVC surfaces (Skowron et al., 2019). We also demonstrated that RCI eradicated not only planktonic cells but also biofilm of *L. monocytogenes* from food contact surfaces i.e., stainless steel, earthenware, rubber, and polypropylene. In this study the lowest bacterial recovery was found for *E. coli*. More bacteria were eliminated from the stainless steel than the rubber surface (Skowron et al., 2018a). The differences between Gram-positive and Gram-negative bacteria in the resistance to photo-catalysis can be associated with the construction of their cell wall (Pal et al., 2005, 2007). On the other hand, Ortega et al. (2007) demonstrated that RCI eliminated around 90% of *S. aureus*, *Bacillus* spp., *E. coli*, *L. monocytogenes*, and *Candida albicans* from the stainless steel.

The first factor that was evaluated was the time exposure to RCI. We have shown that the number of bacteria decreased together with time exposure of RCI, which supports earlier studies. Grinshpun et al. (2007) found that the extension of time exposure from 10 to 30 min increased the reduction of the *Bacillus subtilis* number from 75 to 90%. Ortega et al. (2007) demonstrated that 2 h of RCI treatment allowed a reduction of 90% of bacterial planktonic cells from stainless steel. Our previous study (Skowron et al., 2018a) showed a reduction of 18–99.9% for planktonic cells of *L. monocytogenes* and 3.9–70% for biofilm on food contact surfaces (stainless steel, earthenware, polypropylene) within 30 min. Saini et al. (2014) also assessed the eradication of *L. monocytogenes* from stainless steel and found a reduction of 4.37 log CFU after 15 min of RCI. In turn, Skowron et al. (2018b) revealed the total reduction of *E. coli* and *C. albicans*, and a reduction of 99.9% of *S. aureus*, *Staphylococcus epidermidis* and *Enterococcus faecalis* from the air after a 20-min treatment. This time, however, was probably too short for spore elimination (Skowron et al., 2018b). The demonstrated dependence may have practical applications for the use of RCI technology in the industry, where the operation time is of great importance for the effectiveness of the method used.

In this study, the greatest survival of bacteria exposed to RCI was demonstrated at 4°C, while the lowest survival was noted at 37°C. Better microbicidal activity of RCI was found against stainless steel rather than rubber surfaces. The ozone concentration in the air at 4°C ranged from 0.04 to 0.049 mg/m^3^. There is no data on the impact of temperature on RCI efficacy. Our previous studies revealed the effectiveness of this method against bacteria at room temperature (25°C) (Skowron et al., 2018a). Differences in RCI impact on microbial survival at different temperatures may be related to membrane fluidity and the up-regulation of genes involved in cold adaptation. At low temperatures, genes for fatty acids desaturation, cold shock proteins that serve as RNA chaperones, genes responsible for compatible solutes accumulation are induced and may play a protective role (Shivaji et al., 2010). Furthermore, it has been reported that decrease in temperature-induced gene expression is associated with the oxidative stress and detoxification process (Schade et al., 2004). This may partly explain our results. The above trend is not fully confirmed by the results of Sung et al. (2014). These researchers, assessing the antimicrobial effect of ozone, concluded that effectiveness of ozone in elimination of *E. coli*, *S.* Enteritidis, and *L. monocytogenes* from apple juice was the highest at 50°C, then 4°C, and the lowest at 20°C.

In this study, the influence of the distance from the instrument was also assessed. The lowest number of bacteria was re-isolated from the surfaces at a distance of 0.5 m. The number of recovered bacteria from the surfaces placed directly at the instrument, as well as at a distance greater than 0.5 m, was higher and increased together with the distance. In our previous study, the distance from the RCI instrument was 0.2 m, which allowed a significant reduction of *L. monocytogenes* (Skowron et al., 2018a). Our recent studies (Skowron et al., 2019) proved that a distance of 0.5 m from the RCI cell is sufficient to effectively inactivate *K. pneumoniae* NDM. There is no data on the impact of the distance from the apparatus on the efficiency of photocatalytic processes. However, there are reports indicating the important role of this parameter in the case of other methods of microbial elimination. Research on the effect of the distance on the effectiveness of UV radiation against various species of microorganisms was carried out by Katara et al. (2008). The authors found that the range of 2–16 feet, 8 feet (2.44 m) is the most effective in inactivating *E. coli*. In contrast, Petersson et al. (2014) showed the bactericidal effectiveness of UV light against *Bacillus* spp. and *Clostridium difficile* emitted at a distance of 10 cm. In RCI technology, the factor acting on microbes is activated air, which, depending on the distance and volume of the room, is “diluted” with atmospheric air, losing its effectiveness. Due to the application of RCI in the food industry, the distance from the device is an important factor influencing its practical application.

Our aim was to evaluate the usefulness of RCI as a disinfection method in the food industry. Therefore, we studied the effect of organic contaminants on its bactericidal efficacy. The organic contaminants had a protective effect on the bacteria, which resulted in the decreased reduction of their number in response to RCI. Moreover, such an environment supplied control bacteria, not exposed to RCI, in nutrients leading to their multiplication. This is in agreement with our previous study (Skowron et al., 2018a), where the effectiveness of *L. monocytogenes* inactivation was correlated with the type of organic contaminants. The greatest and the lowest bacterial reduction was also noted for the surfaces contaminated with the carrot pulp and fish pulp, respectively (Skowron et al., 2018a). The RCI was the most efficacious against stainless steel, irrespective of the presence and contamination type. Regardless of the pulp and surface type, the most sensitive to RCI was *E. coli* and the most resistant was *S. aureus*. The greatest bacterial reduction was achieved for the clean surfaces, whereas bacteria survived best in the presence of meat-fish pulp. However, it should be noted that the above studies were carried out using only one reference strain for each of the tested species. Such strains may react differently than environmental strains previously exposed to different adverse conditions. In turn, some authors show such a big variability among the bacterial population that it is difficult to decide if any selected pool of strains reflects the so-called supergenome typical for a given species (Fux et al., 2005). For this reason, these studies should be treated as pilot studies, allowing an evaluation of the impact of various factors of the production environment on the antimicrobial effectiveness of RCI technology. In such a study, the use of commercially available reference strains, with confirmed properties, seems to be the optimal choice. These strains were previously used in other studies by different authors (Reinders et al., 2001; Azizkhani et al., 2013; Lin et al., 2019; Wiktorczyk et al., 2019).

We postulate that further investigation on a larger group of strains, including those derived from the food processing environment, is needed for the application of RCI in the food industry. Our findings can be an initial stage for the research conducted on a much broader technical scale.

## Conclusion

The main objective in this study was to identify the effect of selected environmental factors on the microbiocidal effectiveness of RCI. Among the tested environmental conditions combinations, the greatest number of bacteria was re-isolated at 4°C (distance: 0.5 m, time: 24 h), whereas the lowest number was found at a distance of 0.5 m from the apparatus (temperature 20°C, time: 24 h) and on the surfaces free from organic contaminants. Due to the potential use of technology for the elimination of pathogens in the food industry, the study was to determine the optimal conditions for the device to work effectively against several types of microorganisms. The impact of RCI on microorganisms is not well recognized so far. We have demonstrated that RCI inactivates bacteria in a species-dependent manner, and its efficacy is influenced by environmental conditions. This technology might be a useful disinfection method in the health service and food industry, where effective disinfection is important. However, in the food industry, organic contaminants should be taken into account as a factor limiting disinfection effectiveness. Further studies assessing RCI efficacy against spores, as well as studies on the mechanism of biocidal activity, are needed.

## Data Availability Statement

The raw data supporting the conclusion of this article will be made available by the authors, without undue reservation, to any qualified researcher.

## Author Contributions

KS developed the concept of research and methodology, obtained the equipment, prepared the research stand, analyzed the results, and participated in writing the final version of the manuscript. EW-Z and KG developed the research and methodology, collected the literature, and prepared the original version of the manuscript. JK-P and NW collected statistical analysis of results and interpreted the results. MK was responsible for the comprehensive performance and the development of test results on ozone concentration. ZP, KK-P, and EG-K edited the manuscript. KB and JK conducted the research and collected the results.

## Conflict of Interest

The authors declare that the research was conducted in the absence of any commercial or financial relationships that could be construed as a potential conflict of interest.
